# Predicting and Moderating COVID-Fear and Stress among College Students in Argentina and the USA

**DOI:** 10.3390/ijerph20156510

**Published:** 2023-08-02

**Authors:** Kenneth G. Rice, Fernán Arana, Hannah Wetstone, Michelle Aiello, Barbara Durán

**Affiliations:** 1 Ken Matheny Center for the Study of Stress, Trauma, and Resilience, Department of Counseling and Psychological Services, Georgia State University, Atlanta, GA 30302, USA; hkeenan1@student.gsu.edu (H.W.); maiello1@student.gsu.edu (M.A.); bdennis11@student.gsu.edu (B.D.); 2 Facultad de Psicología, Universidad de Buenos Aires, Buenos Aires C1052, Argentina; fernanarana@psi.uba.ar; 3 National Scientific and Technical Research Council (CONICET), Instituto de Investigaciones en Psicología, Buenos Aires C1033, Argentina

**Keywords:** COVID, stress, college students, emotion regulation, social support

## Abstract

The COVID-19 pandemic negatively affected mental health worldwide and college students were particularly vulnerable to its adverse effects. This longitudinal study was designed to highlight and compare the COVID experiences of college students in Argentina and the USA (N = 361). Specifically, we examined individual factors (gender, emotional regulation, and social support) assessed prior to the pandemic for their role as predictors or moderators of COVID-fear and psychological stress during the first months of the pandemic. The results supported measurement invariance for brief measures of COVID-fear and indicated that, overall, COVID-fear was highest during the second wave of the study (March–April 2020), lowest during the third wave (June 2020), and then rose again during the fourth wave (September 2020). Several interaction effects emerged, revealing important country-level differences in COVID-fear effects for the emotion regulation and social support factors. More so in the Argentina sample than in the USA sample, higher levels of social support at Time 1 were associated with increases in the effect of COVID-fear on stress among students. We discussed the implications of these and other findings for future cross-cultural pandemic-related stress studies.

## 1. Introduction

The coronavirus (COVID-19) produced a global pandemic resulting in significant changes across numerous life domains. State and national leaders around the world implemented social distancing and stay-at-home orders, which prompted the closure of schools and businesses [1,2,3]. College students were uniquely affected, as the shift to online classrooms created disruption in their academic and social lives [4].

Concerns about the virus were high due to its potential to spread without host or victim awareness of initial symptoms and the health risks imposed on vulnerable segments of society. The combination of individuals’ concerns about their own health and the health of their loved ones, restricted social contact, and job loss or insecurity, resulted in a lengthy period of stress exposure for many. Indeed, Daniali et al. documented six major areas of COVID-related concerns across 28 articles that touched on fear and anxiety during the pandemic [5]. Studies indicated that the pandemic had negative effects on the mental health of the general public, and young people and college students were a particularly vulnerable group [6,7]. Psychological difficulties among students have been associated with negative health-related effects, school dropout, and poor occupational attainment [8,9]. Therefore, it seems imperative to investigate the effects of the pandemic on college students more closely, including a focus on factors that might exacerbate or ameliorate adverse pandemic effects.

The current study examined some of the psychological effects of the pandemic on college students and took the novel approach of comparing two countries, Argentina and the USA, that responded in different ways to the onset of the pandemic. Argentina’s government implemented a prolonged period of quarantine from 20 March 2020 to 7 June 2020. Rates of COVID-19 were substantially suppressed until approximately June 2020, during which rates began to accelerate, reaching a peak in July. In contrast to the Argentinian response, the USA did not initially approach the virus as a national problem and instead deferred responsiveness to the virus to states, which in turn resulted in a patchwork of guidance, recommendations, or rules. The national rate of infection began rising in late March and early April 2020. Although rates continued to rise over the subsequent months, there were times of relatively less rapid and relatively more rapid changes [10]. Given these changes, one of the main purposes of the current study was to examine differences in COVID-fear, as well as potential moderators of the relationship between COVID-fear and psychological stress, among college students in the two countries at different time points during the pandemic.

We anticipated that several contemporary issues would likely contribute additional stress to an already difficult time for college students in both countries. Primary examples included the 24-h news cycle, uneven official responses to COVID, concerns about health resources, and academic challenges (e.g., online-only courses). Indeed, studies from early in the pandemic reported an uptick in student stress [11,12]; Tang et al. found that 2.9% of students exceeded clinical cutoffs for PTSD, and 9% were above the cut-off for depression [13]. Similarly, Majumdar et al. found that COVID-related stress was associated with other, more general stress-related problems, such as fatigue, physical symptoms, and depression in college students [14]. We aim to add to this growing body of literature by shedding light on the role of individual factors (e.g., emotional regulation, social support) that may inform college students’ experiences of COVID-fear and stress.

The transactional model of stress [15] is one such approach to understanding stress and provided important conceptual considerations for the current study. The model is particularly useful in the current study because of its emphasis on individual differences in stress perception. That is, the extent to which a potential stressor such as COVID is experienced as stressful depends at least in part on an individual’s perception of COVID. As Lazarus et al. (p. 776) suggested: “No environmental event can be identified as a stressor independently of its appraisal by the person [16]”. The key appraisals are related to a perceived threat (primary appraisal) and the extent to which existing coping resources are perceived as sufficient to help manage the stressor (secondary appraisal). Thus, perceived stress is fundamental, as are characteristics of the person intimately tied to stress appraisals [17,18,19].

Stress appraisals can themselves be predicted by individual characteristics. For example, studies regularly reveal that women report higher levels of perceived stress than reported by men (e.g., [20,21]. Indeed, Bolger and Zuckerman described how individual characteristics can increase the likelihood of exposure to stressors (“differential exposure model”), affect reactivity to stressors (“differential reactivity model”), or affect both exposure and reactivity (“differential exposure/reactivity model”) [22]. For example, differential reactivity could examine whether higher rates of psychological distress reported during COVID (e.g., [23,24]) are associated with specific concerns regarding COVID that are further exacerbated, or buffered, by personal characteristics. As described later in the study, we examined characteristic tendencies involved with emotion regulation as likely to predict stress appraisals, or to exacerbate or buffer the effects stress might have on mental health or other outcomes.

We also considered the perception of social support as an important predictor of COVID-related stress, and a potential moderator of its effects. Both the main and moderating effects of social support have been supported in past studies and reviews (e.g., [25,26]). An important caveat, noted by Cohen and Wills (p. 345), was that “buffering effects are detected primarily when functional support measures provide at least a rough match with the needs elicited by particular types of life stress” [26]. Thus, we were specifically interested in social support that addressed perceptions of available support relevant to issues that might arise within personal, social, and public health concerns surrounding COVID. For that reason, our operationalization of social support included items tapping perceptions of the availability of someone who could care for the individual if bedridden, someone to share worries or fears with, and someone with whom the individual felt close and supported.

Several studies have identified some related factors that may be linked to stress associated with COVID. For example, Liu et al. found that higher levels of COVID-specific worry and loneliness, and lower levels of distress tolerance, resilience, and family support were associated with mental health outcomes in young adults sampled in the USA [27]. In a study on Italian adults, Gullo et al. found that Intolerance of Uncertainty (IU) partially mediated the effect of COVID-fear on depression, stress, and anxiety, and individuals with high IU and expressive suppression showed lower levels of stress [28]. In addition, Zacher and Rudoph conducted a longitudinal study of German participants pre- and post-onset of COVID [29]. They found life satisfaction and positive affect declined during several time periods after COVID was identified as a global pandemic and, in response, Germany began closing daycare centers and imposing restrictions on businesses and social activities. Surprisingly, they also found a significant decline in negative affect during that same time period. In that study, different aspects of stress appraisal and coping strategies had mostly small effects on mental health outcomes.

The fact that several well-performed studies have found relatively modest or negligible links between COVID-related stress and psychological wellbeing suggests that the connection might be a moderated one. Consistent with Bolger and Zuckerman’s model, the current study was designed to examine the possibility that gender, emotion regulation, and social support serve as predictors of COVID-related stress and moderate the effects that such stress might have on psychological outcomes [22]. Consistent with the transactional model, we also considered secondary appraisals of coping resources, specifically the possibility that characteristic tendencies for managing emotional difficulties that were in place prior to the pandemic would predict less COVID-related distress and might buffer the otherwise expected adverse effects of COVID stress. In Nolen-Hoeksema (p. 163), emotion regulation was defined as “the range of activities that allow an individual to monitor, evaluate, and modify the nature and course of an emotional response, in order to pursue his or her goals and appropriately respond to environmental demands” [30]. In the current study, we were particularly interested in both cognitive and emotional aspects of emotion regulation tendencies.

Another important consideration involves the shifting picture of COVID-related issues. For example, depending on the window of time investigated, there might be relatively higher or lower rates of infection, as well as different national or local efforts implemented to reduce risk and transmission. For those reasons, examining the effects and moderators of COVID-related stress might be best accomplished by examining different time frames during the pandemic. Consistent with expectations derived from studies of chronic stress [25], we anticipated that prolonged exposure to the pandemic, with the added features of rebounding and increasing rates of infections at the end of our longitudinal time frame, would contribute to overall higher levels of general psychological stress among students. Thus, we examined predictors of COVID-related stress at each of three different time points, and then further tested the moderating effects of pre-COVID variables on reducing or exacerbating the effects of COVID-fear on general stress at the final time point in our design.

Lastly, we sought to compare COVID-related experiences, and predictive models of COVID stress between students in Argentina and the USA. Where possible, with multi-item measures and after general linguistic, conceptual, and cultural adaptations [31,32], we first examined the comparability of psychometric features using measurement invariance analyses. To our knowledge, this kind of psychometric work has not yet been undertaken with COVID scales, though in fairness, these scales have only recently been reported in the literature.

In summary, the current study was designed to provide some descriptive results relevant to COVID experiences among college students. We also sought to contribute substantive comparisons between Argentina and the USA involving predictors of COVID-fear and moderators of those concerns in predicting general stress. We anticipated that COVID-fear would be predicted by prior personal characteristics known to be linked to stress reactivity (gender, social support), and that those factors as well as potential coping resources (e.g., emotion regulation tendencies) would moderate the effects of COVID fear on psychological stress. Because of the novelty of the COVID pandemic, we did not have specific directional hypotheses regarding the strengths of associations between variables for the two countries.

## 2. Materials and Methods

### 2.1. Participants

At the start of the study (Time 1), participants included 361 undergraduate students from universities in Argentina (ARG) and the United States (USA). Argentinian participants were recruited from a large national university and from several smaller national and private universities. That sample included 218 undergraduate students (71% cisgender female), ranging from ages 18 to 24 (M = 25.87, SD = 5.32). A total of 194 of the 218 students (89%) were Argentinians, and the remainder were from other Latin American countries. Study disciplines included social sciences, sciences, and health professions. Participants from the United States sample included 133 undergraduate students (51% cisgender female) recruited from a large public university in the Southeast. Ages in that sample ranged from 18 to 53 years old (M = 25.08, SD = 5.40). Of the USA participants, students reported the following racial distribution: 18.2% Black, 60.8% White, 9.8% Asian, 20% Hispanic, Latinx, or Spanish ethnicity, and 1% reporting “Other” with an additional 8% of participants not reporting race. Across both samples, all 99.4% of participants selected one of two binary gender options (two responded “decline to answer”), so gender-related analyses are referred to as “gender (binary)” in this article. A total of 72% of ARG participants reported being single and 24% reported they were married/partnered. Just over half of the USA participants (53%) were single, with 28.7% reporting that they were married/partnered. First-generation college students comprised 39.4% of the Argentinian sample and 44.8% of the USA sample. The proportion of employed students was 50% in Argentina and 56.5% in the USA.

### 2.2. Measures

#### 2.2.1. COVID-19 Related Fears

All but one of the scales used in the current study had previously developed English and Spanish language versions. The exception was the [33] SARS Fear Scale (SFS). We followed a linguistic, conceptual, and cultural adaptation process for that scale in which the second author, a bilingual speaker, adapted item content to be consistent with the phrasing and Spanish linguistic preferences of Argentinian speakers. Those items were then back-translated into English and reviewed by the third and fourth authors (also bilingual speakers) for conceptual clarity and consistency with the original items.

The original SFS included 18 items that measured health care providers’ fears of being infected with, infecting others with, or loved ones being infected with severe acute respiratory syndrome (SARS). Additionally, the SFS assessed health care workers’ fears around treating SARS patients. We included 14 out of the 18 SFS items and excluded items related to working in a hospital setting or with SARS patients. We adapted the SFS instructions and items to reflect COVID-19-related fears in a college student population. Specifically, participants were asked to think about the coronavirus (COVID-19) and respond to each item. Two of the original SFS items were modified to reflect college student concerns (i.e., #11 “Dream about myself-family-friends getting infected” and #13 “feel distressed because of academic changes [e.g., all courses having to be taught online]”). Items were rated using a four-point scale: 1 (definitely false), 2 (somewhat false), 3 (somewhat true), and 4 (definitely true), where higher scores suggested greater COVID-19-related fears. In addition, Boyraz et al. recently used a similar rational approach to identify a subset of SFS items that would capture COVID-related worries in a sample of Amazon Mechanical Turk subjects [34]. The current study is the first, to our knowledge, to use the 14-item SFS in both ARG and USA samples. Therefore, we planned factor analyses to extract a reasonable structure to measure COVID-fear in both samples.

#### 2.2.2. Cognitive Emotion Regulation Questionnaire

The short version of the Cognitive Emotion Regulation Questionnaire (CERQ; [35], [36] contains 18 items to measure nine aspects of emotion regulation including Acceptance, Positive Refocusing, Refocusing on Planning, Positive Reappraisal, Putting into Perspective, Rumination, Catastrophizing, Self-blame, and Other Blame. Items are rated using a five-point scale: 1 (almost never), 2 (sometimes), 3 (regularly), 4 (often), and 5 (almost always). The nine emotion regulation subscales are each measured by two items. In Garnefski et al. (p. 1321), two “theoretically more adaptive” and “theoretically less adaptive” factors were also identified that could be measured with the items [37]. The “more adaptive” or “positive-focused” factor consisted of items measuring positive refocusing, positive reappraisal, putting into perspective, refocusing on planning, and acceptance. The “less adaptive” or “negative-focused” factor was measured with items tapping rumination, self-blame, blaming others, and catastrophizing. We modeled the two-factor approach in the current study.

#### 2.2.3. MOS Social Support Survey

The MOS Social Support Survey (MOS; [38]) is a six-item questionnaire used to measure individuals’ perceptions of the psychological and material resources available in their interpersonal relationships (e.g., [39,40,41]). Items are scored using a five-point scale: 1 (none of the time), 2 (a little of the time), 3 (some of the time), 4 (most of the time), and 5 (all of the time), with higher scores representing a greater sense of social support within relationships. The reliability and validity of MOS scores have been supported in college student samples [42].

#### 2.2.4. Perceived Stress Scale

The 10-item version of the Perceived Stress Scale (PSS; (e.g., [43,44,45] was designed to assess the extent to which individuals regard their lives as unpredictable, overwhelming, and out of control. Items are rated on a five-point scale: 0 (never), 1 (almost never), 2 (sometimes), 3 (fairly often), and 4 (very often), indicating how often respondents have had specific stress-related thoughts or feelings over the past month. Higher scores on the PSS reflect more psychological stress. The reliability and validity of PSS scores have been supported in studies of university students [46,47].

### 2.3. Procedure

We recruited undergraduate college students from a large southeastern university in the United States (USA) and a large set of universities in Argentina (ARG). The USA students were recruited from a research participation pool and received credit toward a research requirement during the first semester of the study (Times 1 and 2). For Times 3 and 4, participants received $15 USD in compensation. The Argentinian participants were recruited through media, student blogs, and social networks. Nearly half of the students were from a central national university and the rest were recruited from a variety of national and private universities across the entire country. Financial compensation was the same for the ARG sample at Times 3 and 4 ($15 USD), but they did not receive research credits for their earlier participation. Participants completed measures through an online survey tool (Qualtrics). Several items were added to the surveys to screen for inattentive or careless responses [48]. Participants who did not correctly answer all validity questions had their data excluded from the study.

We collected data at four time periods from January 2020 to September 2020. Time 1 measures of predictors occurred from January to February 2020. During this time, several countries across the world were seeing rising case counts, but COVID-19 had not yet garnered worldwide attention. No cases had been reported in Argentina, and the first cases were reported in the USA in late February. Time 2 occurred from March to April 2020. In Argentina, the first COVID-19 death was documented in early March, and on 11th March the World Health Organization (WHO) declared the outbreak a pandemic. In late March, the number of COVID-19 cases in the USA reached the highest in the world. Time 3 occurred in June 2020, when the count of global COVID-19 cases surpassed 10 million. Both countries were seeing increasing rates of cases and deaths. Time 4 occurred in early September 2020, when case counts were continuing to rise in both countries. In Argentina, President Fernández extended a previously implemented lockdown by another month, and in the USA, the death count from COVID-19 reached 200,000.

### 2.4. Data Analysis

Analyses were conducted with IBS SPSS Version 26 (2019) and Mplus Version 8.4 [49] using the robust MLR estimator. Full information maximum likelihood procedures were implemented by default in Mplus. Measurement models were analyzed with confirmatory factor analyses and evaluated using common fit indices. Based on Brown and Hu and Bentler, acceptable model fit could produce a comparative fit index (CFI) in the 0.90–0.95 range, a root mean square error of approximation (RMSEA) near or less than 0.06, and a standardized root mean square residual (SRMR) of 0.08 or less [50,51]. Measurement invariance analyses were conducted to ensure at least metric invariance of indicators between the two countries.

Apart from COVID-fear, all of the measures used in the current study had previously been used in other research. However, most of the scales had not been used in cross-national comparisons. Therefore, preliminary measurement analyses were conducted to confirm whether prior factor structures for the measures could be supported in this two-country study and, if not, that reasonable alternative structures could be developed from the items. Here, we briefly summarize the approach and analyses. More details are provided in the Supplementary Materials. In general, for each scale, we began with its previously published structure and scoring, and used confirmatory factor analyses (CFAs) and measurement invariance tests to confirm the scale’s utility for the current study. Most of those analyses did not support the expected structure for between-country comparisons, therefore, we relied on modifications based on CFAs or exploratory factor analyses (EFAs) to inform item sets and factors. In several instances, EFAs based on separate analyses for the two countries were compared, and items that strongly loaded on factors in both countries were then selected as the measures. Most of the measures required either some modest adjustments or reconsideration based on the EFAs. Ultimately, we were able to compile three to five items from each subscale or scale to serve as reasonable operationalizations of the study constructs.

Longitudinal measurement invariance was examined for COVID-fear, with additional tests to evaluate structural parameters (factor means, variances, and covariances) over time. COVID-fear at each time point was regressed on the set of Time 1 variables to evaluate differential exposure. Interaction models were used to evaluate differential reactivity. Specifically, we tested whether Time 1 factors moderated the effects between Time 4 COVID-fear and Perceived Stress and whether those effects varied by country.

## 3. Results

### 3.1. Preliminary Analyses

Sample sizes across time points varied as a result of the longitudinal design (attrition) and different sampling prompts. Students who participated at Time 1 were invited to participate at Time 2. Those who did not participate in Time 2 were not invited to complete measures at Time 3. To increase the sample size during a time of increasing COVID rates in both countries, all students who completed Time 1 were invited to participate in Time 4. In the combined Argentina and USA sample, 361 participated at Time 1, 257 (71.2% of the original sample) participated at Time 2, 205 (57%) participated at Time 3, and 168 (46.5%) participated at Time 4. Of the total number of students who participated at Time 1, 131 (36%) participated at all time points.

Prior to major analyses, we evaluated whether participant retention was a function of study variables. First, we used chi-square analyses and *t*-tests to examine whether participant demographics (gender and age) predicted participation at Times 2, 3, and 4. Participant retention at Time 2 through to Time 4 was not associated with gender (*p*s > 0.204) nor age (*p*s > 0.426) across the two countries. There were no significant differences between participants from the two countries in their retention at Times 2 and 4 (*p*s < 0.163). However, there was a significant difference in participation at Time 3 between the two countries (*p* < 0.001), such that a higher proportion of students in Argentina were retained (N = 145, 67% of the Time 1 sample) compared to students in the USA (N = 60, 42% of the Time 1 sample). Then, we used logistic regression analyses to examine whether participant attrition over time was predicted by Time 1 scores on the main study variables. There was not a significant association between participant attrition and Time 1 variables (Positive Reframing, Rumination, and Social Support), *p*s > 0.257. Depending on the analysis and subset of variables, covariance coverage ranged from 0.44 to 1.0. Little’s MCAR test based on Time 1 predictors, Times 2, 3, and 4 COVID-fear, and Time 4 Perceived Stress was consistent with missingness on variables being random rather than systematic, χ^2^ (49, N = 361) = 33.57, *p* = 0.955. Based on the retention analyses, data were assumed to be missing at random and potentially missing completely at random. This permitted the use of full information maximum likelihood to estimate parameters based on all available information, a preferred method for handling missing data [52].

We also tested and supported measurement models for our major predictors. The details of these analyses are shown in the Supplementary Materials. Of note were analyses involving the CERQ. Following Garnefski et al., we initially created adaptive and less adaptive emotion regulation factors with items representing positive-focused (e.g., positive reappraisal) or negative-focused (e.g., rumination) emotion regulation approaches [37]. That model did not fit the data well in both countries, even after several modifications. An EFA and follow-up CFAs supported two factors. One factor consisted of items originally measuring acceptance, positive reappraisal, positive reframing, and putting into perspective. That factor was labeled “Positive Reframing”. The second factor contained two items measuring rumination and one measuring refocus on planning. That factor was labeled “Rumination”. Thus, the initial major analyses consisted of four predictors: gender (binary designation of cisgender women and cisgender men), Positive Reframing, Rumination, and Social Support.

### 3.2. COVID-Fear: Cross-Sectional and Longitudinal Measurement Invariance

The factor structure for the SFS in Ho et al. as not supported in the current study (e.g., CFI = 0.862 for the USA sample) [33]. Based on EFA results and item-factor comparisons between the two countries, we identified five items that reflected COVID-related stress and fears, such as fear of infection and infecting others, and concerns that the virus would get out of control. At Time 2, this single-factor model fit the data well for both ARG (e.g., CFI = 0.933) and USA (e.g., CFI = 0.937) samples. Separate measurement invariance analyses within each time point supported cross-sectional metric and partial scalar invariance for this measure as well as the other scales and item sets used in the current study (see Table 1). Within Times 2, 3, and 4, there were no factor mean differences in COVID-fear between ARG and the USA; *p*s = 0.747, 0.159, and 0.525, respectively.

The two samples were combined for longitudinal invariance tests. Longitudinal metric and partial scalar measurement invariance were supported in those analyses (see Table 1). Structural invariance tests indicated no significant differences in factor variances (*p* = 0.209) or covariances (*p* = 0.766) over time. That is, individual differences in COVID-fear were equivalent over time, as was the relative stability of COVID-fear. Freely estimated factor correlations ranged from 0.51 to 0.59. Factor means were significantly different across time (*p* = 0.0003), suggesting changes in absolute stability. Across time points for both countries, the highest level of COVID-fear was at Time 2, followed by the lowest level at Time 3, then a slightly and significantly higher level at Time 4.

### 3.3. Differential Exposure: Predictors of COVID-Fear

Given the shifting patterns in COVID-19 infections over time, and perhaps some corresponding desensitization, it may not be surprising that there would be changes in the degree of concerns from COVID-19. These differences suggested that we might examine within-time snapshots of COVID-fear and whether variations in those levels were related to our Time 1 predictors. We examined these effects using cross-lagged panel models to further account for the autoregressive effects of COVID-fear. That is, these analyses examined the effects of the predictors after partialling the effects of prior COVID-fear. Because SEM approaches to these models produced estimation problems, likely owing to model complexity and modest sample sizes, factor scores derived from metric invariance measurement models were used in these and later analyses.

An initial model allowed freely estimated paths from predictors to each COVID-fear outcome at Time 2, 3, and 4, for both the ARG and USA samples. That model was tested against a model in which those paths were constrained to be invariant between the samples. There was not a significant difference between the freely estimated and constrained invariant models for the block of predictors, Δχ^2^ (12, N = 360) = 12.04, *p* = 0.443. Framed differently, country did not moderate the effect of the five Time 1 predictors on COVID-fears at each of the subsequent three time points. Because the effects were not significantly different for the two countries, the samples were combined for a reanalysis of the predictors. The block of predictors revealed nonsignificant effects in accounting for variation in COVID-fear at Time 2 (R^2^ = 0.04, *p* = 0.104), Time 3 (R^2^ = 0.03, *p* = 0.217), and Time 4 (R^2^ = 0.01, *p* = 0.448). Thus, the combination of predictors did not account for significant variation in COVID-fear at each of those three time points, indicating no support for differential exposure.

### 3.4. Differential Reactivity: Moderating the Effects of COVID-Fear on Psychological Stress

Because other studies have documented psychological risks associated with the pandemic, we also examined whether more specific COVID-fears were associated with general psychological stress, and whether that effect was moderated by any of the Time 1 predictors. Furthermore, given our interest in the similarities and differences between ARG and the USA, we included country as a second moderator in those models. We used Mplus to test full models that included each three-way interaction (Time 4 COVID-fear × Time 1 Predictor × Country) and all two-way interactions and conditional direct effects, with Time 4 Perceived Stress as the dependent variable. The results are summarized in Table 2.

### 3.5. Gender (Binary)

The overall model explained 22% of the variance in Time 4 Perceived Stress (*p* = 0.001). The three-way interaction of COVID-fear × Country was not significant, *p* = 0.282. However, the COVID-fear × Country two-way interaction was significant, B = −0.39, SE = 0.15, *p* = 0.01. As might be expected, this two-way interaction emerged in several of the other analyses involving Time 1 predictors and, therefore, is only summarized in this section. Controlling for other effects in the model, for ARG, the association between COVID-fear and Perceived Stress was positive and significant, (B = 0.41, SE = 0.09, *p* < 0.001). For the USA, that effect was not significant (B = 0.13, SE = 0.09, *p* = 0.145). The conditional effect for gender was also significant (*p* = 0.034), consistent with women reporting higher levels of perceived stress compared with men. In sum, gender did not moderate the effect of COVID-fear on Perceived Stress, but country did.

### 3.6. Positive Reframing

The full model involving the CERQ Positive Reframing factor explained 22% of the variance in Perceived Stress (*p* < 0.001). The three-way interaction of COVID-fear × Positive Reframing × Country was significant, *p* = 0.003. We further explored the interaction by calculating the simple effects of COVID-fear predicting PSS at low (16th percentile) and high (84th percentile) levels of Positive Reframing for the two countries. For ARG, the association between COVID-fear and Stress was positive and significant at both levels (*p*s < 0.001), suggesting no substantial moderating effect; the effect (slope) was only slightly higher when Positive Reframing was high (B = 0.51, SE = 0.11, *p* < 0.001) compared to when Positive Reframing was low (B = 0.37, SE = 0.11, *p* < 0.001). In contrast, the effect between COVID-fear and Stress for the USA sample was stronger when Positive Reframing was low (B = 0.57, SE = 0.11, *p* < 0.001) than it was at high levels of Positive Reframing (B = 0.11, SE = 0.14, *p* = 0.403). These results suggested that the levels of Perceived Stress were relatively unaffected by COVID-fear in the USA sample for those with high levels of Positive Reframing. Such an effect would be consistent with a stress-reducing role of Positive Reframing in the USA sample.

**Figure 1:**
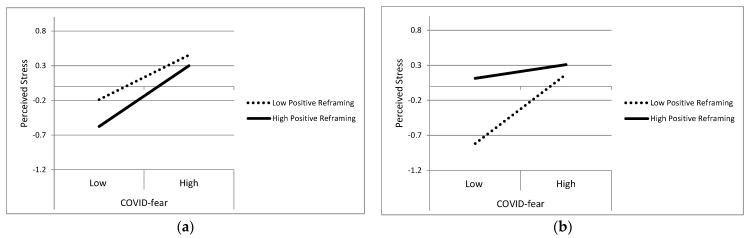
Three-way interaction of COVID-fear × Positive Reframing × Country Predicting Perceived Stress. Panel (**a**) shows the COVID-fear × Positive Reframing interaction for the Argentina sample and panel (**b**) displays the interaction for the USA sample.

The plot of expected Stress scores at low (16th percentile) and high (84th percentile) levels of Positive Reframing and COVID-fear revealed a more nuanced interpretation (see Figure 1). The effect in ARG seemed generally consistent with a main effect such that higher Stress was associated with higher levels of COVID-fear, with no substantial variation in that effect attributable to Positive Reframing. In the USA, students who had low levels of Positive Reframing reported the least Stress when COVID-fear was low, but under high COVID-fear, their level of Stress was dramatically higher. Interestingly, a higher level of Stress was predicted for USA students who had high Positive Reframing, regardless of their COVID-fear level. Indeed, the predicted Stress score for those with low Positive Reframing and high COVID-fear was comparable to students with high Positive Reframing (see Figure 1). In isolation, their significant and positive slope reflected a stress-exacerbating effect among the USA students with low Positive Reframing, but results also were consistent with high and stable rates of stress for students endorsing high Positive Reframing. In contrast, for ARG and across COVID-fear levels, the most stressed students were those endorsing low levels of Positive Reframing, and both groups experienced greater stress at higher levels of COVID-fear.

### 3.7. Rumination

The full model including the other CERQ factor of Rumination accounted for significant overall variation in Perceived Stress (R^2^ = 0.18, *p* < 0.001). The three-way interaction of COVID-fear × Rumination × Country was not significant, *p* = 0.198. There was a trend effect for COVID-fear × Rumination (*p* = 0.081). Controlling for the other variables in the model, at the low level of Rumination, COVID-fear had a stronger effect on Perceived Stress (B = 0.41, SE = 0.10, *p* < 0.001) than it did at the high level of Rumination (B = 0.28, SE = 0.10, *p* = 0.006). Thus, across both countries, higher levels of Rumination had a stress-dampening effect on COVID-fear.

### 3.8. Social Support

**Figure 2:**
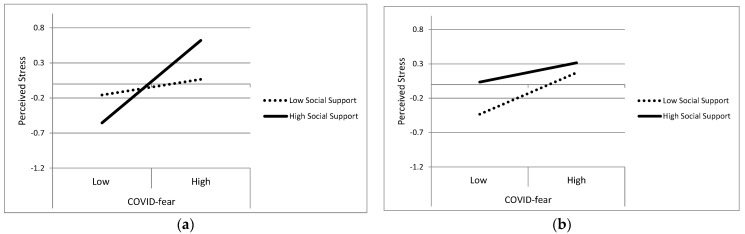
Three-way interaction of COVID-fear × Social Support × Country Predicting Perceived Stress. Panel (**a**) shows the COVID-fear × Social Support interaction for the Argentina sample and panel (**b**) displays the interaction for the USA sample.

The model with Time 1 Social Support explained 23% of Time 4 Perceived Stress variation, *p* < 0.001. The three-way effect of COVID-fear × Social Support × Country was significant, *p* = 0.022. The effect of COVID-fear on Perceived Stress varied at high and low levels of Social Support for the two countries. The evaluation of simple effects indicated that, for ARG, there was a stronger association between COVID-fear and Stress when Social Support was high (B = 0.71, SE = 0.12, *p* < 0.001) than when Social Support was low (B = 0.10, SE = 0.12, *p* = 0.392). For the USA, COVID-fear had a stronger association with Perceived Stress when Social Support was low (B = 0.36, SE = 0.14, *p* = 0.01) than when Social Support was high (B = 0.15, SE = 0.23, *p* = 0.501). Thus, for ARG, higher levels of Social Support had a stress-exacerbating effect on the COVID-fear to Perceived Stress association. For the USA, the opposite effect was observed such that higher levels of COVID-fear were associated with higher levels of Perceived Stress, but only when Social Support was low. Of additional note, however, was that in the USA, students reporting higher Social Support also reported high levels of Perceived Stress. In ARG, only when COVID-fear was high were those students also likely to report higher Stress than the low Social Support students. These effects are displayed in Figure 2.

### 3.9. Summary

Cross-sectional measurement invariance analyses yielded substantial support for relatively brief measures of psychological risk factors and support for the use of the COVID-fear indicators in longitudinal research. There were strong but not completely overlapping associations between COVID-fear along with fluctuating levels at the different times points in the study. This meant that concerns about COVID at one point in time were likely to predict concerns at a later time point, but there was some subsequent shifting in the relative concerns. Patterns of mean differences revealed an early peak in COVID-fear at Time 2 followed by a substantial decline at Time 3, which was then followed by an uptick in COVID-fear level at Time 4, but one that was still below the original threat perceived at Time 2. Recall that Time 2 was the first point at which we collected the COVID-fear data.

The differential reactivity (moderator) models helped to expand our understanding of COVID-fear beyond the stability and shifting levels of concerns, and beyond the negligible effects observed from the differential exposure models. Positive Reframing as an emotion regulation approach did not substantially alter the otherwise significant effect of COVID-fear effects on psychological stress for the ARG students. Interestingly, results in the USA sample were consistent with a COVID-fear stress-exacerbating effect when students had low Positive Reframing, but also revealed generally high levels of Perceived Stress for students with high Positive Reframing, regardless of the COVID-fear level. In contrast, both countries had comparable effects involving the COVID-fear and Rumination interaction. In general, higher levels of Rumination were associated with a weakened association between COVID-fear and Stress. Finally, in ARG, the effect of COVID-fear on general psychological stress was exacerbated more so for those who reported high levels of Social Support than for those with low support. In the USA, the opposite effect emerged such that there were no differences in Perceived Stress as a function of COVID-fear when students had high Social Support. Instead, COVID-fear only exacerbated Stress for those with low Social Support.

## 4. Discussion

### 4.1. Discussion

The COVID-19 pandemic contributed to major, global disruptions in social behavior, economic indicators, and psychological wellbeing [53]. College students were particularly affected due to shifts in academic and social aspects of college life (e.g., shift to online classes, the closing of campus residence halls, elimination of or delays to graduation and athletic events), and studies show that college students’ mental health was severely affected [54]. Stress from the pandemic can be further attributed to the lack of a vaccine in the early stages of the pandemic, the possibility of transmission from asymptomatic individuals, the politicization of the health crisis, and persistent media reports, all of which contributed to classic features of stress such as uncertainty, uncontrollability, and chronicity of the stressors [55]. There were also substantial variations in official community and national responses to the virus, likely contributing to fluctuations in health indicators (e.g., infections, hospitalizations, death) and perceptions regarding the threats posed by the pandemic. Prior theory [15] and considerable prior evidence (e.g., [56]) also point to personal or psychological factors that can affect stress appraisals and ultimately stress-related consequences. The main purpose of the current study was to examine the effects of personal characteristics assessed prior to the intensification of the pandemic in predicting subsequent COVID-related concerns and psychological stress among college students later during the pandemic. We additionally focused on risk for general perceived stress later in the pandemic and examined predictor and moderator models in two countries that implemented different approaches to the pandemic. Although several recent studies have examined the stress reactions and related consequences of COVID, to our knowledge, none have examined the effects that prior personal characteristics, such as gender, emotion regulation, and perceived social support have had on COVID-related experiences and mental health among college students.

An important set of psychometric findings in the current study supported a brief measure of COVID-fear that could be used for cross-sectional and longitudinal studies in both countries. The five items were derived from [33] the SARS Fear Scale and tapped concerns related to becoming infected, infecting others, and worries that the respondent’s family would become infected, along with the feeling that “the virus will get out of control”. The levels of those concerns shifted over time in both countries, with the relatively highest levels reported early in the pandemic. The lowest levels of COVID-fear were reported at the start of June 2020, during what appeared to be, in retrospect, a slight uptick in new cases in both countries. The last data collection episode in September 2020 coincided with a dramatic rate of increasing cases in ARG and the start of what would become another upsurge in the USA during the subsequent months. COVID-fear showed a relative increase over the June level at that time as well but did not reach the same intensity as March–April. Several factors might have played a role in the initial peak and generally suppressed post-April levels of COVID-fear. The early peak possibly was due to the novelty of the situation combined with relatively little actionable news, concerns about controllability, and unknowns regarding risk [57,58]. As time unfolded, substantially high-risk rates emerged for elderly and medically-compromised individuals, and although younger college students could become infected, their risk for dramatic adverse consequences was shown to be much lower than for the elderly. Thus, concerns about infection and controllability could have diminished [59]. By September, the mild escalation in COVID fears in students from both countries could be explained through local rather than global lenses. In the USA, more optimistic expectations about returning to some level of normalcy on campus were modestly reduced when students were confronted with local, campus-specific increased rates of infection and the ongoing need to implement social distancing and other safety procedures [1]. For Argentinian students, nevertheless, there was no room for optimism about returning to regular classes. By September, the national lockdown had reached 180 days, wherein academic activities were exclusively provided online. Indeed, the increment in COVID fears in Argentinian students could be more related to the uncertainty about the completion of the lockdown and a combination of increasing feelings of jadedness and reduced feelings of calm, both probably triggered by the social consequences of the national lockdown [60]. Alternatively, for both countries, the generally lower level of COVID-fear in June and September could be an accurate reflection of students taking necessary precautions and believing in their efficacy to reduce risk. Other aspects of COVID might be stressful for students but perhaps a more direct link between COVID itself and stress or wellbeing is more challenging to detect. Another consideration is that the stress associated with a national event such as COVID may be much broader than COVID fear and the general stress examined in this study. In fact, researchers have explored what is now being called COVID stress syndrome, which includes four factors: fear of the dangerousness of COVID, worry about the socioeconomic costs of COVID, xenophobic fears that foreign individuals are spreading the virus, traumatic stress symptoms related to COVID (i.e., nightmares), and COVID-related compulsive checking and reassurance seeking [61]. Future studies might examine how these symptoms differ in their predictors and prospective outcomes among students.

Note that, despite overall shifts in overall level, the relative stability of COVID-fear was moderately high (0.51–0.59 range). Thus, those students with higher levels of COVID-fear at one point in time were generally also likely to be at the higher end of the distribution of scores at subsequent time points. To be sure, the correlations were high but not so high as to consider perfect predictability, so some students with earlier higher levels of fear shifted lower in the distribution, and some with earlier low levels shifted higher, but in general, earlier COVID-fear was a good predictor of later COVID-fear. The size of those effects was in keeping with a state-based rather than trait indicator of concerns, likely providing further evidence of the utility of the scale. More stable personal and interpersonal characteristics were used in subsequent analyses to attempt to explain variance in shifting perceptions of COVID-fear.

Surprisingly, the Time 1 variables that have been shown in other studies to be important predictors of stress reactivity were, in the current study, inconsequential in their prediction of later COVID-fear. Perhaps the challenge of stress detection directly associated with COVID described earlier might also help explain their negligible effect. That is, the emphasis on infection-related and virus-controllability concerns might not be as stressful for students as other, more proximal stressors in their daily lives (e.g., financial concerns, difficulty performing well in online courses, seeking employment). Future studies might explore these aspects of student life.

Although the direct effects of predictors on COVID-fear were not significant, we also examined other ways in which the predictors and COVID-fear might be important. More specifically, we evaluated the additive and interactive associations of the Time 1 variables and COVID-fear in predicting more pervasive general psychological stress. We also extended those models to incorporate country effects, allowing us to evaluate whether the Time 1 predictor variables operated in a different way in interaction with COVID-fear for students in ARG and the USA. Several effects emerged that involved differences and some similarities between the countries.

As a starting point, it should be noted that each of the separate models explained substantial and significant variance (18–23%) in psychological stress during the September (Time 4) evaluation. Although substantial, the individual differences variables have often been shown to be strong predictors of stress and psychological distress. Furthermore, analyses were based on factor scores derived from CFA measurement models, which very likely reduced error in the analyses and sharpened the confidence that true score relations were being tested.

Of additional note were the moderator effects, especially those supporting the three-way interaction of a Time 1 predictor × COVID-fear × country. Those interactions revealed important country-level and potential cultural differences in COVID-fear effects for the emotion regulation and social support factors, in particular. For example, positive reframing might ordinarily be considered an adaptive emotion regulation strategy; some mixed support for that expectation was found in the USA sample, though it was also the case that higher positive reframing students reported higher levels of stress regardless of COVID-fear. For the ARG sample, high levels of positive reframing may have worsened rather than reduced the effect COVID-fear was having on general stress. The ARG students with low use of positive reframing seemed better off, at least in terms of the effect COVID-fear was having on their stress.

Opposite effects emerged for the ARG and USA samples in the stress-related effects of positive reframing tendencies, and such effects seem to highlight the importance of considering contextual and cultural contexts, as well as meaning-making that occurs when participants read questionnaire items and weigh the adaptiveness or maladaptiveness of coping strategies. There were also curious effects involving the overall level of stress for students who might otherwise be thought of as using adaptive emotion regulation strategies. Thus, understanding the variations in effects we detected can be informed by recalling the operationalization of the emotion regulation constructs and how those items might tap into different experiences and cultural factors in the two countries.

In the USA, the items for positive reframing seemed likely to tap into the need for understanding the positive and growth-related potential in difficult situations, although results suggested that reframing tended to stabilize stress at a higher level regardless of COVID-fear. In contrast, low levels of reframing were associated with a rapid strengthening of the association between COVID-fear and stress, consistent with stress exacerbation. Because general stress tended to be higher for the students endorsing high Positive Reframing, it seems unreasonable to interpret positive reframing as having a stress-reducing effect for USA students. Perhaps the item content for Positive Reframing should be considered in the narrative for those students to understand these implications. The items tapped acceptance of the situation, resilience or a steeling effect (I will be stronger), perspective (things could be worse), and refocusing (think about something nice). Although ordinarily such a narrative, at least in the USA context, would be linked to better problem-solving and emotional wellbeing [62], the current study found high levels of acknowledged stress for those students. In the USA, the surreal nature of the COVID pandemic for many young people, coinciding with a stressful election year, may have contributed to these strategies being associated with higher stress regardless of actual COVID-fear a student reported. In fact, the least stressed students when COVID-fear was low were those who had the lowest levels of Positive Reframing. However, those students were also the most at risk of a rapid rise in stress with higher COVID-fear, consistent with a stress-exacerbating effect for Positive Reframing. Recall that the stress exacerbating effects of low Positive Reframing seemed to do little more than rapidly help those students match the higher stress levels of their high Positive Reframing counterparts. Perhaps reframing and acceptance approaches, as assessed with the subset of CERQ items used in the present study, are not advisable for managing stress during a public health emergency in the USA.

There was not substantial support for a moderating effect of Positive Reframing in the ARG sample. Instead, COVID-fear predicted higher levels of general stress, with stress rising at about the same rate for those with high and low levels of Positive Reframing. The narrative for the ARG students might be, “I can attempt to think differently about this situation, but the reality is that there is much to be fearful of with COVID and thinking differently does not reduce the stress and concerns I have about my health and the health of others close to me”.

Rumination effects were comparable between the two countries, which was consistent with other previous cross-cultural findings, though with a different measure of rumination [61]. Higher levels of rumination had stress-reducing effects on COVID-fear. That effect suggested that the factor might be tapping “deliberate rumination” [63] or more positive aspects of reflection and introspection than negative perseverative tendencies often associated with psychological distress (e.g., [64,65]). Indeed, rumination about uncontrollable events seems to have a less deleterious effect than a rumination on controllable events, because when people ruminate on events, such as an earthquake or a pandemic, there is more opportunity to cope with self-distance, which has some ego-protecting features, particularly when previous levels of pandemic anxiety were high [66]. Future studies, especially those that intervene in on deliberate rumination during similar major events, could strengthen that inference.

Effects involving social support varied by country and were especially intriguing. In general, social support is considered a major element in stress reduction or stress management [24]. The current findings present a more complicated story involving social support that indicates its effects might vary depending on the nature of the stressor, and the country and cultural context within which one resides. In some respects, the USA sample results were consistent with the general understanding of social support as a stress buffer; for those with high levels of support, COVID-fear had no effect on psychological stress. It was only for those with low support that COVID-fear was a strong predictor of stress. However, consistent with the results involving Positive Reframing, the most stressed students tended to be those who reported the highest levels of social support. To be sure, based on the nonsignificant slope, their stress level did not seem to be worsened as a function of COVID-fear, but was nonetheless at a higher level compared with the low social support students. Note that the items comprising social support in the current study included some that, under other circumstances, would be unlikely to pose extreme adverse health consequences for the providers of support (e.g., “Someone to take you to the doctor if you needed it”; “Someone to do something enjoyable with”). Similarly, social support probably works, in general, to reduce stress when the provider of support is not experiencing the same stressor as the person in need of the support. Obviously, that is not the case during a pandemic. Such nuances seem worth further examination in additional studies.

The effects in ARG also were contrary to the general narrative for social support but in ways that differed from the USA. COVID-fear effects on psychological stress were significantly stronger for students with high levels of social support, and trivial when social support was low. Those results suggested that social support might have exacerbated rather than buffered the effects of COVID-fear on psychological stress. One possible explanation is that, compared to those with low support, ARG students with strong support connections may feel like they have more to lose from a virus that must be managed in large part by reducing social contact. In contrast to the USA, students in Argentina typically live with their parents while attending college. Furthermore, being part of a more collectivistic society than USA students, Argentinian students tend to value, and suffer, from their social bonds, particularly their bonds with relatives [67]. Such students reside in existing social networks of support. In fact, for students in ARG who are likely to be living with parents and other family members, reducing social contact between family members may be a tall and unrealistic expectation. Against the backdrop of other relational benefits, however, such students might also have more opportunities to communicate with others about COVID and share their concerns, potentially and mutually exacerbating the effects of COVID-fear [68]. This possibility seems consistent with the summary in Rachman regarding fear acquisition by the transmission of verbal information. In the modern era of technology and social media, there may be a high likelihood that information spread through connections people have with family and friends could have been fear-inducing, especially early in the pandemic [69]. The combination of proximity and collectivistic attitudes might have heightened fear of infecting parents and other relatives. Such an explanation is consistent with results in other research, indicating the main concern in a large Argentinian sample was fear of the family being infected [70,71]. These dynamics might also help explain the effects involving ARG students with low social support who, based on the nonsignificant slope, seemed to have a general stress level that was unaffected by COVID-fear. Perhaps, for those students, COVID-fear and its social implications are inconsequential in large part because they do not have anything to lose in terms of social bonds or more direct interpersonal implications of COVID given their already existing sense of social disconnection.

### 4.2. Implications

The findings of this study have implications for college students and universities navigating major health-related disruptions. Although findings should be interpreted with caution considering the correlational design, it seems that, broadly, positive reframing as an emotion regulation strategy may not be useful for managing stress during a public health emergency with so many unknowns. Alternative coping strategies such as deep breathing or meditation may be more beneficial and can be recommended by college counselors working with students in Argentina and the U.S. In fact, Green et al. found that mindfulness meditation reduced the worsening of mental health due to the COVID-19 pandemic among engineering students in the U.S. [72], and a past study found that the combination of deep breathing, relaxation response, meditation, and guided imagery techniques with cognitive behavioral techniques helped reduce stress among undergraduate students in Argentina [73].

Additionally, the finding that social support seemed to have an exacerbating effect of COVID-fear on stress in Argentina may suggest that students could benefit from increased awareness about the effects of significant others on their stress levels during broad-reaching health-related events. Counselors and universities in Argentina can help with building students’ awareness through psychoeducational methods, working with students on setting boundaries with significant others where possible, and bolstering students’ individual coping strategies. In the U.S., social support appears to be helpful but not for students who are highly stressed. Thus, counselors and universities can help those students by assessing stress levels, building awareness, and bolstering students’ individual coping strategies.

## 5. Conclusions

In summary, the current study combined measurement development and substantive research questions to examine the prediction of COVID-fear, and the moderation of those concerns in predicting later psychological stress among college students in Argentina and the USA. Psychometric evaluations supported a concise set of items that could be reliably used in ARG (Spanish) and the USA (English) to track COVID-fear, and findings revealed country-level differences in COVID-fear effects. Social support seemed to only have an exacerbating effect of COVID-fear on stress for students in Argentina, and less so for students in the USA. Future studies, especially those that intervene in social support, would strengthen that inference. Additional work is warranted to deepen the understanding of the role that emotion regulation and coping strategies have for college students in managing disruptions brought about by a global pandemic.

## Figures and Tables

**Table 1:** Summary of goodness of fit indices and model comparisons for COVID-fear invariance tests.

Model	χ^2^	df	Δχ^2^	Δdf	*p*	CFI	∆CFI	MNCI	ΔMNCI	RMSEA	90% CI	SRMR
Cross-Sectional (Country) Invariance												
*Time 2*												
Configural	25.04	10				0.934		0.979		0.110	0.057, 0.165	0.044
Metric	25.20	14	1.22	4	0.875	0.951	0.017	0.984	0.005	0.080	0.023, 0.130	0.051
Scalar	72.33	18	51.57	4	<0.0001	0.761	−0.190	0.961	−0.058	0.156	0.119, 0.194	0.108
Partial Scalar ^a^	31.30	16	6.37	2	0.041	0.933	−0.018	0.979	−0.006	0.088	0.040, 0.133	0.064
*Time 3*												
Configural	15.65	10				0.974		0.994		0.074	0.000, 0.142	0.036
Metric	16.55	14	1.00	4	0.909	0.988	0.014	0.986	0.008	0.042	0.000, 0.109	0.042
Scalar	59.25	18	49.99	4	<0.0001	0.808	−0.180	0.904	−0.090	0.150	0.109, 0.193	0.124
Partial Scalar ^a^	17.24	16	0.57	2	0.751	0.994	0.006	0.997	0.003	0.028	0.000, 0.098	0.043
*Time 4*												
Configural	8.26	10				1.000		1.005		0.000	0.000, 0.104	0.029
Metric	12.06	14	3.94	4	0.414	1.000	0.000	1.006	0.001	0.000	0.000, 0.093	0.047
Scalar	33.47	18	20.51	4	<0.0001	0.920	−0.800	0.954	0.102	0.102	0.045, 0.156	0.084
Partial Scalar ^a^	17.50	16	5.09	2	0.079	0.992	−0.008	0.995	−0.011	0.034	0.000, 0.110	0.051
Longitudinal Invariance												
Configural	101.43	72				0.964		0.947		0.039	0.019, 0.056	0.057
Metric	111.46	80	9.82	8	0.278	0.962	−0.002	0.943	−0.004	0.028	0.019, 0.054	0.062
Scalar	130.66	88	19.01	8	0.015	0.948	−0.014	0.924	−0.019	0.042	0.026, 0.057	0.071
Partial Scalar ^a^	114.29	86	2.64	6	0.852	0.966	0.004	0.949	0.006	0.035	0.014, 0.051	0.063

Note. Item-to-factor models were based on preliminary measurement analyses described in the Supplementary Materials. CFI = Comparative Fit Index. RMSEA = Root Mean Square Error of Approximation. 90% CI = confidence interval for RMSEA. SRMR = Standardized Root Mean Square Residual. MNCI = McDonald’s noncentrality index. Δχ^2^ based on the Yuan-Bentler scaling correction. ^a^ two freed intercepts.

**Table 2:** Time 1 variables moderating effect of Time 4 COVID-fear on perceived stress.

Primary Moderator/Predictors	B	SE	*p*
Gender (Binary)			
COVID-fear	0.34	0.10	<0.0001
Gender	−0.41	0.19	0.034
Country	0.001	0.15	0.995
COVID-fear X Gender	0.17	0.20	0.399
COVID-fear X Country	−0.39	0.15	0.01
Gender X Country	0.19	0.25	0.440
COVID-fear X Gender X Country	0.28	0.26	0.282
Positive Reframing			
COVID-fear	0.44	0.09	<0.0001
Positive Reframing	−0.17	0.09	0.047
Country	−0.04	0.11	0.683
COVID-fear X Positive Reframing	0.09	0.09	0.312
COVID-fear X Country	−0.10	0.13	0.429
Positive Reframing X Country	0.51	0.13	<0.0001
COVID-fear X Positive Reframing X Country	−0.38	0.13	0.003
Rumination			
COVID-fear	0.41	0.08	0.000
Rumination	0.00	0.09	0.963
Country	−0.01	0.11	0.908
COVID-fear X Rumination	−0.16	0.09	0.081
COVID-fear X Country	−0.20	0.15	0.177
Rumination X Country	0.09	0.16	0.554
COVID-fear X Rumination X Country	0.26	0.21	0.198
Social Support			
COVID-fear	0.39	0.08	0.000
Social Support	0.05	0.10	0.621
Country	0.02	0.11	0.834
COVID-fear X Social Support	0.34	0.10	0.001
COVID-fear X Country	−0.13	0.13	0.328
Social Support X Country	0.14	0.17	0.405
COVID-fear X Social Support X Country	−0.45	0.20	0.022

Note. *p*-values are based on two-tailed *z* statistics.

## Data Availability

Data available on request.

## Supplementary Materials

The following supporting information can be downloaded at: https://www.mdpi.com/article/10.3390/ijerph20156510/s1. Exploratory factor analyses, final cross-sectional measurement invariance results, and Supplementary Tables S1–S4. References [74,75,76,77,78,79,80,81,82,83] are cited in Supplementary Materials.

## References

[1] Smalley A. National Conference of State Legislatures. https://www.ncsl.org/education/higher-education-responses-to-coronavirus-covid-19.

[2] Coronavirus: Suspensión de Clases Presenciales 14 Días Consecutivos a Partir Del 16 de Marzo. Argentina.gob.ar. https://www.argentina.gob.ar/noticias/coronavirus-suspension-de-clases-presenciales-14-dias-consecutivos-partir-del-16-de-marzo.

[3] Argentina.gob.ar. https://www.argentina.gob.ar/sites/default/files/acerca_de_los_90.000_comercios_que_supuestamente_cerraron_0.pdf.

[4] Birmingham W.C., Wadsworth L.L., Lassetter J.H., Graff T.C., Lauren E., Hung M. (2021). COVID-19 lockdown: Impact on college students’ lives. J. Am. Coll. Health.

[5] Daniali H., Martinussen M., Flaten M.A. (2023). A global meta-analysis of depression, anxiety, and stress before and during COVID-19. Health Psychol..

[6] Wang C., Pan R., Wan X., Tan Y., Xu L., McIntyre R.S., Choo F.N., Tran B., Ho R., Sharma V.K. (2020). A longitudinal study on the mental health of general population during the COVID-19 epidemic in China. Brain. Behav. Immun..

[7] Coelho C.M., Suttiwan P., Arato N., Zsido A.N. (2020). On the nature of fear and anxiety triggered by COVID-19. Front. Psych..

[8] Hjorth C.F., Bilgrav L., Frandsen L.S., Overgaard C., Torp-Pedersen C., Nielsen B., Bøggild H. (2016). Mental health and school dropout across educational levels and genders: A 4.8-year follow-up study. BMC Public Health.

[9] Slominski L., Sameroff A., Rosenblum K., Kasser T.I.M. (2011). Longitudinal predictors of adult socioeconomic attainment: The roles of socioeconomic status, academic competence, and mental health. Dev. Psychopathol..

[10] Centers for Disease Control and Prevention. https://www.cdc.gov/museum/timeline/covid19.html..

[11] Huckins J.F., da Silva A.W., Wang W., Hedlund E., Rogers C., Nepal S.K., Wu J., Obuchi M., Murphy E.I., Meyer M.L. (2020). Mental health and behavior of college students during the early phases of the COVID-19 pandemic: Longitudinal smartphone and Ecological Momentary Assessment Study. J. Med. Internet Res..

[12] Son C., Hegde S., Smith A., Wang X., Sasangohar F. (2020). Effects of COVID-19 on college students’ mental health in the United States: Interview survey study. J. Med. Internet Res..

[13] Tang W., Hu T., Yang L., Xu J. (2020). The role of alexithymia in the mental health problems of home-quarantined university students during the COVID-19 pandemic in China. Personal. Individ. Differ..

[14] Majumdar P., Biswas A., Sahu S. (2020). COVID-19 pandemic and lockdown: Cause of sleep disruption, depression, somatic pain, and increased screen exposure of office workers and students of India. Chronobiol. Int..

[15] Lazarus R.S., Folkman S. (1984). Stress, Appraisal, and Coping.

[16] Lazarus R.S., DeLongis A., Folkman S., Gruen R. (1985). Stress and adaptational outcomes: The problem of confounded measures. Am. Psychol..

[17] Bibbey A., Carroll D., Roseboom T.J., Phillips A.C., Rooij S.R. (2013). Personality and physiological reactions to acute psychological stress. Int. J. Psychophysiol..

[18] Chida Y., Hamer M. (2008). Chronic psychosocial factors and acute physiological responses to laboratory-induced stress in healthy populations: A quantitative review of 30 years of investigations. Psychol. Bull..

[19] Dickerson S.S., Kemeny E. (2004). Acute stressors and cortisol responses: A theoretical integration and synthesis of laboratory research. Psychol. Bull..

[20] Barbosa-Leiker C., Kostick M., Lei M., McPherson S., Roper V., Hoeskstra T., Wright B. (2013). Measurement Invariance of The Perceived Stress Scale and Latent Mean Differences Across Gender and Time. Stress Health J. Int. Soc. Investig. Stress.

[21] Nolen-Hoeksema S. (2001). Gender differences in depression. Curr. Dir. Psychol. Sci..

[22] Bolger N., Zuckerman A. (1995). A framework for studying personality in the stress process. J. Pers. Soc. Psychol..

[23] Ettman C.K., Abdalla S.M., Cohen G.H., Sampson L., Vivier P.M., Galea S. (2020). Prevalence of depression symptoms in US adults before and during the COVID-19 pandemic. JAMA.

[24] Fitzpatrick K.M., Harris C., Drawve G. (2020). Living in the midst of fear: Depressive symptomatology among US adults during the COVID-19 pandemic. Depress. Anxiety.

[25] Cohen S. (2004). Social relationships and health. Am. Psychol..

[26] Cohen S., Wills T.A. (1985). Stress, social support, and the buffering hypothesis. Psychol. Bull..

[27] Liu Y., Gayle A.A., Wilder-Smith A., Rocklöv J. (2020). The reproductive number of COVID-19 is higher compared to SARS coronavirus. J. Travel Med..

[28] Gullo S.S., Gelo O.C.G., Bassi G., Lo Coco G., Lagetto G., Esposito G., Pazzagli C., Salcuni S., Freda M.F., Mazzeschi C. (2022). The role of emotion regulation and intolerance to uncertainty on the relationship between fear of COVID-19 and distress. Curr. Psychol..

[29] Zacher H., Rudolph C.W. (2020). Individual differences and changes in subjective wellbeing during the early stages of the COVID-19 pandemic. Am. Psychol..

[30] Nolen-Hoeksema S. (2012). Emotion regulation and psychopathology: The role of gender. Annu. Rev. Clin. Psychol..

[31] Maxwell B., Martin M.O., Kelly D.L. (1996). Translation and cultural adaptation of the survey instruments. Third International Mathematics and Science Study (TIMSS), Technical Report, Volume I: Design and Development.

[32] Muñiz J., Hambleton R.K. (1996). Directrices para la traducción y adaptación de los tests. Papeles Psicólogo.

[33] Ho S., Cook K.V., Chen Z.J., Kurniati N.M.T., Suwartono C., Widyarini N., Wong P.T.P., Cowden R.G. (2022). Suffering, psychological distress, and well-being in Indonesia: A prospective cohort study. Stress Health.

[34] Boyraz G., Legros D.N. (2020). Coronavirus disease (COVID-19) and traumatic stress: Probable risk factors and correlates of posttraumatic stress disorder. J. Loss Trauma.

[35] Garnefski N., Kraaij V. (2006). Cognitive emotion regulation questionnaire-development of a short 18-item version (CERQ-short). Personal. Individ. Differ..

[36] Medrano L.A., Moretti L., Ortiz A., Pereno G. (2015). Validación del Cuestionario de Regulación Emocional Cognitiva en Universitarios de Córdoba. Argent. Psykhe.

[37] Garnefski N., Kraaij V., Spinhoven P. (2001). Negative life events, cognitive emotion regulation and emotional problems. Personal. Individ. Differ..

[38] Sherbourne C.D., Stewart A.L. (1991). The MOS social support survey. Soc. Sci. Med..

[39] Gómez-Campelo P.E., Pérez-Moreno E.M., Burgos-Lunar C., Bragado-Álvarez C., Jiménez-García R., Salinero-Fort M.Á. (2014). Psychometric properties of the eight-item modified Medical Outcomes Study Social Support Survey based on Spanish outpatients. Qual. Life Res. Int. J. Qual. Life Asp. Treat. Care Rehabil..

[40] Holden L., Lee C., Hockey R., Ware R.S., Dobson A.J. (2014). Validation of the MOS Social Support Survey 6-item (MOS-SSS-6) measure with two large population-based samples of Australian women. Qual. Life Res..

[41] Rodríguez S., Rodríguez E., Camelo H. (2007). Validación Argentina del cuestionario MOS de apoyo social percibido. Psicodebate.

[42] Giangrasso B., Casale S. (2014). Psychometric properties of the medical outcome study social support survey with a general population sample of undergraduate students. Soc. Indic. Res..

[43] Baik S.H., Fox R.S., Mills S.D., Roesch S.C., Sadler G.R., Klonoff E.A., Malcarne V.L. (2019). Reliability and validity of the Perceived Stress Scale-10 in Hispanic Americans with English or Spanish language preference. J. Health Psychol..

[44] Cohen S., Kamarck T., Mermelstein R. (1983). A global measure of perceived stress. J. Health Soc. Behav..

[45] Cohen S., Williamson G., Spacapan S.S., Oskamp S. (1988). Perceived stress in a probability sample of the United States. The Claremont Symposium on Applied Social Psychology. The Social Psychology of Health.

[46] Roberti J.W., Harrington L.N., Storch E.A. (2006). Further psychometric support for the 10-item version of the perceived stress scale. J. Coll. Couns..

[47] Smith K.J., Rosenberg D.L., Haight T.G. (2014). An assessment of the psychometric properties of the Perceived Stress Scale-10 (PSS 10) with business and accounting students. Account. Perspect..

[48] Curran P.G. (2016). Methods for the detection of carelessly invalid responses in survey data. J. Exp. Soc. Psychol..

[49] Muthén L.K., Muthén B.O. (1998). Mplus User’s Guide.

[50] Brown T.A. (2015). Confirmatory Factor Analysis for Applied Research.

[51] Hu L.T., Bentler P.M. (1999). Cutoff criteria for fit indexes in covariance structure analysis: Conventional criteria versus new alternatives. Struct. Equ. Model. Multidiscip. J..

[52] Schlomer G.L., Bauman S., Card N.A. (2010). Best practices for missing data management in counseling psychology. J. Couns. Psychol..

[53] Salari N., Hosseinian-Far A., Jalali R., Vaisi-Raygani A., Rasoulpoor S., Mohammadi M., Rasoulpoor S., Khaledi-Paveh B. (2020). Prevalence of stress, anxiety, depression among the general population during the COVID-19 pandemic: A systematic review and meta-analysis. Glob. Health.

[54] Reyes-Portillo J.A., Warner C.M., Kline A.E., Bixter M.T., Chu B.C., Miranda R., Nadeem E., Nickerson A., Peralta A.O., Reigada L. (2022). The psychological, academic, and economic impact of COVID-19 on college students in the epicenter of the pandemic. Emerg. Adulthood.

[55] Sapolsky R.M. (2004). Social status and health in humans and other animals. Annu. Rev. Anthropol..

[56] Cooper C., Quick J.C. (2017). The Handbook of Stress and Health: A Guide to Research and Practice.

[57] Banerjee D. (2020). The COVID-19 outbreak: Crucial role the psychiatrists can play. Asian J. Psychiatry.

[58] Mueller A.L., McNamara M.S., Sinclair D.A. (2020). Why does COVID-19 disproportionately affect older people?. Aging.

[59] Observatorio Psicología Social Aplicada (2020). Crisis Coronavirus Estudio Nº 12: 180 Días De Cuarentena: Salud Mental, Economía y Gestión Política.

[60] Taylor S., Landry C.A., Paluszek M.M., Fergus T.A., McKay D., Asmundson G.J. (2020). COVID stress syndrome: Concept, structure, and correlates. Depress. Anxiety.

[61] Nolen-Hoeksema S., Wisco B.E., Lyubomirsky S. (2008). Rethinking rumination. Perspect. Psychol. Sci..

[62] Arana F.G., Rice K.G. (2020). Cross-cultural validity of the Ruminative Responses Scale in Argentina and the United States. Assessment.

[63] García F.E., Duque A., Cova F. (2017). The four faces of rumination to stressful events: A psychometric analysis. Psychol. Trauma Theory Res. Pract. Policy.

[64] Aldao A., Nolen-Hoeksema S., Schweizer S. (2010). Emotion-regulation strategies across psychopathology: A meta-analytic review. Clin. Psychol. Rev..

[65] Ottaviani C., Medea B., Lonigro A., Tarvainen M., Couyoumdjian A. (2015). Cognitive rigidity is mirrored by autonomic inflexibility in daily life perseverative cognition. Biol. Psychol..

[66] Kross E., Ayduk O., Olson J.M. (2017). Self-distancing: Theory, research, and current directions. Advances in Experimental Social Psychology.

[67] Chiou J.S. (2001). Horizontal and vertical individualism and collectivism among college students in the United States, Taiwan, and Argentina. J. Soc. Psychol..

[68] Dillard J.P., Yang C., Li R. (2018). Self-regulation of emotional responses to Zika: Spiral of fear. PLoS ONE.

[69] Rachman S. (1991). Neo-conditioning and the classical theory of fear acquisition. Clin. Psych. Rev..

[70] Estudio Tiara (2020). Primer Avance de Resultados. http://repositorio.cedes.org/handle/123456789/4534.

[71] Facio A., Resett S., Micocci F., Mistrorigo C. (2007). Emerging Adulthood in Argentina: An Age of Diversity and Possibilities. Child Dev. Perspect..

[72] Green J., Huberty J., Puzia M., Stecher C. (2021). The effect of meditation and physical activity on the mental health impact of COVID-19–related stress and attention to news among mobile app users in the United States: Cross-sectional survey. JMIR Ment. Health.

[73] Iglesias S.L., Azzara S., Argibay J.C., Arnaiz M.L., de Valle Carpineta M., Granchetti H., Lagomarsino E. (2012). Psychological and physiological response of students to different types of stress management programs. Am. J. Health Promot..

[74] Arnett A.B., Pennington B.F., Willcutt E., Dmitrieva J., Byrne B., Samuelsson S., Olson R.K. (2012). A cross-lagged model of the development of ADHD inattention symptoms and rapid naming speed. J. Abnorm. Child Psychol..

[75] Dimitrov D.M. (2010). Testing for factorial invariance in the context of construct validation. Meas. Eval. Couns. Dev..

[76] Grice J.W. (2001). Computing and evaluating factor scores. Psychol. Methods.

[77] Kam C., Morin A.J.S., Meyer J.P., Topolnytsky L. (2016). Are commitment profiles stable and predictable? A latent transition analysis. J. Manag..

[78] Kang Y., McNeish D.M., Hancock G.R. (2016). The role of measurement quality on practical guidelines for assessing measurement and structural invariance. Educ. Psychol. Meas..

[79] McDonald R.P., Marsh H.W. (1990). Choosing a multivariate model: Noncentrality and goodness of fit. Psychol. Bull..

[80] Meade A.W., Johnson E.C., Braddy P.W. (2008). Power and sensitivity of alternative fit indices in tests of measurement invariance. J. Appl. Psychol..

[81] Morin A.J., Marsh H.W. (2015). Disentangling shape from level effects in person-centered analyses: An illustration based on university teachers’ multidimensional profiles of effectiveness. Struct. Equ. Model..

[82] Morin A.J., Boudrias J.S., Marsh H.W., Madore I., Desrumaux P. (2016). Further reflections on disentangling shape and level effects in person-centered analyses: An illustration exploring the dimensionality of psychological health. Struct. Equ. Model..

[83] Sass D.A. (2011). Testing measurement invariance and comparing latent factor means within a confirmatory factor analysis framework. J. Psychoeduc. Assess..

