# Supplementary material for: Predicting and Moderating COVID-Fear and Stress among College Students in Argentina and the USA
Source: Int J Environ Res Public Health. 2023 Aug 2;20(15):6510. doi: 10.3390/ijerph20156510 (PMC10418435; doi:10.3390/ijerph20156510)
Supplement: Supplementary file 1 [file ijerph-20-06510-s001.zip › ijerph-2406018-supplementary.pdf]

Supplemental Material

General Overview

The starting point for measurement analyses was to conduct a confirmatory factor analysis (CFA) based on existing published guidance for factor structure. Separate CFAs were conducted for each sample (ARG, USA). M*plus* with the MLR estimator was used for all CFAs. If both CFAs supported the hypothesized structure, multiple groups CFAs were planned as the next step to evaluate measurement invariance. Because none of the existing a priori measurement structures reached the second step, the strategy was changed. We conducted separate exploratory factor analyses (EFAs) for each sample and set of items from each scale. The EFAs were conducted in SPSS using the principal axis factoring option and, where needed for multiple factor models, PROMAX rotation was used. We then identified items in the separate EFAs that had relatively higher factor loadings for both samples. Discussions regarding item retention centered on interpretability in both the ARG and USA contexts. Ultimately, we selected at least four indicators to represent each construct of interest. Those reduced item sets were subjected to multiple groups CFAs and a series of cross-sectional measurement models to evaluate configural, metric, and scalar invariance. Metric invariance was necessary to have confidence in sample comparisons involving strengths of associations between the constructs. Scalar invariance was deemed not as essential for the substantive analyses. As described in the main article text, we also conducted longitudinal measurement

invariance analyses for the COVID-fear scale.

Different measurement models were evaluated using common fit indices: Comparative Fit Index (CFI), Root Mean Square Error of Approximation (RMSEA), Standardized Root Mean Square Residual (SRMR), and McDonald's noncentrality index (MNCI; McDonald & Marsh, 1990). Nested models (more constrained minus less constrained) in the invariance tests were compared using differences in the scaling-corrected CHI-SQ, ΔCFI (decrease > .002; Meade et al., 2008), and ΔMNCI (decrease > .007; Kang et al., 2016). Simulation studies have supported the MNCI as especially robust at detecting non-invariance in a variety of different study contexts (Kang et al., 2016). Following recommendations from Dimitrov (2010) and Sass (2011), instances of non-invariance would be further explored through descriptive statistics to evaluate the practical extent of non-invariance. We also considered revising models if non-invariance was detected (e.g., allowing for partial invariance).

Measurement Models

Short Cognitive Emotion Regulation Questionnaire (CERQ; Garnefski & Kraaij, 2006)

The 18-item CERQ was originally designed to measure nine factors with two items to measure each factor. Additional research on the CERQ supported an adaptive or "positive-focused" factor and a less adaptive or "negative-focused" factor (Garnefski et al., 2001, p. 1321). We initially used CFA to evaluate the two-factor model. The positive factor consisted of the 10 items that reflected positive refocusing, positive reappraisal,

putting into perspective, refocus on planning, and acceptance. The negative factor consisted of 8 items reflecting rumination, self-blame, blaming others, and catastrophizing. That model did not fit the data well in the ARG (CFI = 0.546) or USA (CFI = 0.694) samples. A scree plot and parallel analysis based on an EFA of the items supported a three-factor solution in both samples. Factor loadings for that solution appear in Table S1. The third factor in the ARG solution had only two items with substantial loadings that did not cross-load on another factor. Similarly, the third factor in the USA sample also had several items cross-loading with other factors, and those were not the same cross-loading items as in the ARG sample. In an effort to extract a parsimonious structure that might perform well in both samples, we eliminated the cross-loadings items and conducted additional EFAs. Parallel analysis again supported three factors but again the third factor could be indicated by only two items in both samples. We reanalyzed the data extracting two factors and a more interpretable solution emerged. Factor loadings for that solution are displayed in Table S2. Based on the original item-to-factor designations on the CERQ, Factor 1 in both samples consisted of items measuring acceptance, positive reappraisal, putting into perspective, refocus on planning, and positive refocusing, all of which reflected more positive or adaptive cognitive emotion regulation tendencies. In contrast, the five indicators of Factor 2 represented other-blame, rumination, and refocus on planning, or for the most part, what might be considered less adaptive emotion regulation approaches.

Table S1

Exploratory Factor Analysis of CERQ Items: Promax Rotated Factor Loadings for the Three-Factor Solution

	Argentina			USA		
Item	F1	F2	F3	F1	F2	F3
I think that I have to accept that this has happened	**0.64**	0.12	-0.15	**0.51**	0.09	-0.15
I often think about how I feel about what I have experienced	0.18	**0.50**	0.17	0.09	**0.73**	-0.11
I think I can learn something from the situation	**0.59**	0.11	-0.07	**0.49**	0.10	-0.12
I feel that I am the one who is responsible for what has happened	0.01	**0.54**	**-0.38**	0.23	**0.57**	0.04
I think that I have to accept the situation	**0.55**	0.03	-0.13	**0.48**	0.05	-0.07
I am preoccupied with what I think and feel about what I have experienced	-0.04	**0.67**	0.12	-0.08	**0.65**	0.00
I think of pleasant things that have nothing to do with it	**0.44**	-0.21	0.18	**0.46**	-0.06	0.23
I think that I can become a stronger person as a result of what has happened	**0.66**	0.13	0.06	**0.54**	0.10	-0.29
I keep thinking about how terrible it is what I have experienced	-0.15	**0.65**	0.21	-0.23	**0.48**	**0.45**
I feel that others are responsible for what has happened	0.01	0.21	**0.69**	0.06	-0.06	**0.69**
I think of something nice instead of what has happened	**0.53**	-0.09	0.26	**0.55**	-0.08	**0.36**

I think about how to change the situation	0.22	**0.42**	0.05	0.16	**0.41**	0.01
I think that it hasn't been too bad compared to other things	**0.45**	-0.11	-0.08	**0.59**	-0.19	0.26
I think that basically the cause must lie within myself	0.03	**0.58**	**-0.35**	0.06	**0.59**	0.04
I think about a plan of what I can do best	**0.40**	0.01	0.15	**0.49**	0.25	-0.04
I tell myself that there are worse things in life	**0.54**	0.01	0.01	**0.59**	0.01	0.10
I continually think how horrible the situation has been	-0.16	**0.51**	0.27	-0.13	**0.37**	**0.40**
I feel that basically the cause lies with others	0.07	0.09	**0.74**	-0.02	0.07	**0.66**

Table S2

Exploratory Factor Analysis of CERQ Items: Promax Rotated Factor Loadings for the Two-Factor Solution

	Argentina		USA	
Item	F1	F2	F1	F2
I think that I have to accept that this has happened	**0.70**	-0.06	**0.58**	-0.13
I think that I can become a stronger person as a result of what has happened	**0.67**	0.14	**0.60**	-0.16
I think I can learn something from the situation	**0.63**	0.04	**0.58**	-0.10
I think that I have to accept the situation	**0.61**	-0.12	**0.55**	-0.06
I tell myself that there are worse things in life	**0.50**	0.02	**0.50**	0.18

I think that it hasn't been too bad compared to other things	**0.41**	-0.08	**0.48**	0.14
I think about a plan of what I can do best	**0.37**	0.13	**0.47**	0.21
I think of pleasant things that have nothing to do with it	**0.33**	-0.06	**0.37**	0.10
I feel that others are responsible for what has happened	-0.15	**0.60**	-0.07	**0.61**
I feel that basically the cause lies with others	-0.11	**0.52**	-0.13	**0.63**
I am preoccupied with what I think and feel about what I have experienced	-0.04	**0.52**	-0.05	**0.46**
I often think about how I feel about what I have experienced	0.18	**0.50**	0.10	**0.45**
I think about how to change the situation	0.23	**0.35**	0.14	**0.41**

A subsequent CFA based on eight indicators for an adaptive coping factor and five indicators produced a poor fit for both the ARG (CFI = 0.579) and USA (CFI = 0.695) samples. Item content and comparability between the two cultural contexts was then considered, along with modification indices, size of factor loadings from both the EFAs and CFAs, and redundant content with some items. Several additional factor analyses were conducted with different subsets of items from the two factors. Ultimately, we settled on four items that generally presented positive reframing, acceptance, and perspective, and three that primarily represented rumination or reflection. The four Positive Reframing indicators were, "I think that I have to accept that this has

happened," "I think that I can become a stronger person as a result of what has happened," "I think of something nice instead of what has happened," and "I tell myself that there are worse things in life." The three Rumination items were, "I often think about how I feel about what I have experienced," "I am preoccupied with what I think and feel about what I have experienced," and "I think about how to change the situation." Other-blame items had low loadings and contributed to poor model fit and were excluded. The 7-item CERQ fit the data reasonably well in both the ARG (CFI = 0.916), and USA (CFI = 0.928) samples. Cross-sectional measurement invariance analyses confirmed at least metric (factor loading) invariance between the countries (see Table S4).

MOS Social Support Survey (Sherbourne & Stewart, 1991)

The six MOS items were initially subjected to a CFA that resulted in poor fit for the ARG sample (CFI = 0.689) but excellent fit for the USA sample (CFI = 0.965). Results suggested that item 4 ("Someone to take you to the doctor if you needed it") could be excluded because of low loading and redundant content with item 3 ("Someone to help you if you were confined to bed"). The five item MOS fit the data extremely well in ARG (CFI = 0.989) and the USA (CFI = 0.980) and that item set was advanced for invariance testing. Results displayed in Table S4 supported metric invariance, although to support the metric model, a minor adjustment was required involving correlated error terms for items 1 ("Someone to share your most private worries and fears with")

and 2 (“Someone to turn to for suggestions about how to deal with a personal problem”) but only in the ARG sample. The remaining three items on the scale were: “Someone to help you if you were confined to bed,” “Someone to do something enjoyable with,” and “Someone to love and make you feel wanted.”

Perceived Stress Scale (Cohen et al., 1983)

At Time 4, the 10-item PSS was administered, and CFAs of the original single-factor model produced less than desired fit for the ARG sample (CFI = 0.886) and the USA sample (CFI = 0.692). Although most of the 10 items had good factor loadings for the ARG, in the USA, six items had good loadings and four were extremely low. These differences in fit and loadings led us to conduct EFAs for both samples. Scree plot and parallel analyses for the two samples supported a single factor solution in the ARG sample but a two-factor solution in the USA. Because of our interest in comparing general and overall psychological stress in the two samples, we examined both the one- and two-factor models for a core set of indicators that loaded onto a comparable factor in both groups. The factor loadings for those solutions appear in Table S3.

Across the different solutions and samples, we identified these four items as representing a general psychological stress factor with strong indicators in both groups: “How often have you felt difficulties were piling up so high that you could not overcome them?” “How often have you been upset because of something that happened unexpectedly?” “How often have you felt nervous and ‘stressed’?” and

"How often have you felt that you were unable to control the important things in your life?" Measurement invariance analyses based on these four items also supported their metric invariance (see Table S4).

Table S3

Factor Loadings for the One-Factor and Two-Factor (PROMAX) Solutions Based on Exploratory Factor Analysis of PSS Items

	Argentina			USA		
	1-Factor	2-Factor		1-Factor	2-Factor	
Item	1F1	2F1	2F2	1F1	2F1	2F2
How often have you felt difficulties were piling up so high that you could not overcome them?	**0.77**	**0.62**	0.23	**0.65**	**0.65**	0.11
How often have you been upset because of something that happened unexpectedly?	**0.74**	**0.73**	0.02	**0.68**	**0.68**	0.06
How often have you felt nervous and "stressed"?	**0.72**	**0.82**	-0.12	**0.52**	**0.52**	-0.05
How often have you felt that you were unable to control the important things in your life?	**0.72**	**0.81**	-0.12	**0.57**	**0.56**	0.10
How often have you felt that you were on top of things?	**0.68**	**0.60**	0.12	-0.03	-0.05	**0.52**
How often have you been angered because of things that happened that were outside of your control?	**0.68**	**0.53**	0.24	**0.50**	**0.53**	-0.26
How often have you felt confident about your ability to handle your personal problems?	**0.61**	**0.52**	0.15	0.04	0.03	0.42

How often have you found that you could not cope with all the things that you had to do?	**0.60**	**0.69**	-0.12	**0.76**	**0.75**	-0.01
How often have you felt that things were going your way?	**0.52**	**0.41**	0.17	0.01	-0.02	**0.60**
How often have you been able to control irritations in your life?	**0.46**	-0.10	**0.97**	0.11	0.10	**0.47**

Factor Scores

Factor scores were saved based on the final metric invariance measurement models and used in the major substantive analyses (Kam et al., 2016; Morin & Marsh, 2015; Morin et al., 2016). As a compromise between the use of raw scores and structural equation modeling, using factor scores is more rigorous than using observed raw scores (with error) and less demanding in parameter estimation than would have been the case with SEM and modest sample sizes (see Arnett et al., 2012; Kam et al., 2016; Morin & Marsh, 2015; Morin et al., 2016). Determinacy estimates (validity coefficients) or the correlation between the estimated and true factor scores were high (ranging from .80 to .93) and provided additional confidence in their use (Grice, 2001).

Table S4

Cross-Sectional Measurement Invariance Results Comparing Argentina and the USA

Scale/Model	χ^2	df	$\Delta\chi^2$	Δdf	*p*	CFI	ΔCFI	RMSEA	[90% CI]	SRMR	MNCI	ΔMNCI
Cognitive Emotion Regulation Questionnaire (CERQ; 2-Factor, 7 items)												
Configural	46.85	26				0.923		0.067	0.035,0.098	0.057	0.970	
Metric	54.98	31	8.28	5	.141	0.911	-.012	0.066	0.036,0.094	0.065	0.966	-.004
Scalar	104.01	36	52.95	5	<.0001	0.747	-.164	0.104	0.081,0.127	0.090	0.907	-.059
Medical Outcomes Survey-Social Support Scale (MOS; 1-Factor, 5 items)												
Configural	14.23	9				0.988		0.057	0.000,0.110	0.024	0.993	
Metric	21.26	13	7.28	4	.122	0.981	-.007	0.060	0.000,0.140	0.055	0.989	-.004

Scalar	40.38	17	20.86	4	.0003	0.946	-.035	0.088	0.053,0.123	0.082	0.968	-.021
Perceived Stress Scale (PSS; 1-Factor, 4 items)												
Configural	6.45	4				0.985		0.088	0.000,0.208	0.028	0.992	
Metric	7.31	7	1.16	3	.764	0.998	.013	0.024	0.000,0.142	0.042	0.999	.007
Scalar	34.66	10	27.50	3	<.0001	0.085	-.148	0.177	0.115,0.243	0.116	0.024	-.075

References

Arnett, A. B., Pennington, B. F., Willcutt, E., Dmitrieva, J., Byrne, B., Samuelsson, S., & Olson, R. K. (2012). A cross-lagged model of the development of ADHD inattention symptoms and rapid naming speed. *Journal of Abnormal Child Psychology*, *40*(8), 1313-1326. https://doi.org/10.1007/s10802-012-9644-5

Cohen, S., Kamarck, T., & Mermelstein, R. (1983). A global measure of perceived stress. *Journal of Health and Social Behavior*, *24*(4), 385-396. https://doi.org/10.2307/2136404

Dimitrov, D. M. (2010). Testing for factorial invariance in the context of construct validation. *Measurement and Evaluation in Counseling and Development*, *43*(2), 121-149. https://doi.org/10.1177/0748175610373459

Garnefski, N., Kraaij, V., & Spinhoven, P. (2001). Negative life events, cognitive emotion regulation and emotional problems. *Personality and Individual Differences*, *30*(8), 1311-1327. https://doi.org/10.1016/S0191-8869(00)00113-6

Garnefski, N., & Kraaij, V. (2006). Cognitive emotion regulation questionnaire-development of a short 18-item version (CERQ-short). *Personality and Individual Differences*, *41*(6), 1045-1053. https://doi.org/10.1016/j.paid.2006.04.010

Grice, J. W. (2001). Computing and evaluating factor scores. *Psychological Methods*, *6*(4), 430-450. https://doi.org/10.1037/1082-989X.6.4.430

Kam, C., Morin, A. J. S., Meyer, J. P., & Topolnytsky, L. (2016). Are commitment profiles stable and predictable? A latent transition analysis. *Journal of Management, 42(6),*

1462-1490. https://doi.org/10.1177/0149206313503010

Kang, Y., McNeish, D. M., & Hancock, G. R. (2016). The role of measurement quality on practical guidelines for assessing measurement and structural invariance. *Educational and Psychological Measurement, 76(4)*, 533-561. https://doi.org/10.1177/0013164415603764

McDonald, R. P., & Marsh, H. W. (1990). Choosing a multivariate model: Noncentrality and goodness of fit. *Psychological Bulletin, 107(2),* 247-255. https://doi.org/10.1037/0033-2909.107.2.247

Meade, A. W., Johnson, E. C., & Braddy, P. W. (2008). Power and sensitivity of alternative fit indices in tests of measurement invariance. *Journal of Applied Psychology*, *93*(3), 568-592. https://doi.org/10.1037/0021-9010.93.3.568

Morin, A. J., & Marsh, H. W. (2015). Disentangling shape from level effects in person-centered analyses: An illustration based on university teachers' multidimensional profiles of effectiveness. *Structural Equation Modeling: A Multidisciplinary Journal*, *22*(1), 39-59. https://doi.org/10.1080/10705511.2014.919825

Morin, A. J., Boudrias, J. S., Marsh, H. W., Madore, I., & Desrumaux, P. (2016). Further reflections on disentangling shape and level effects in person-centered analyses: An illustration exploring the dimensionality of psychological health. *Structural Equation Modeling: A Multidisciplinary Journal*, *23*(3), 438-454. https://doi.org/10.1080/10705511.2015.1116077

Sass, D. A. (2011). Testing measurement invariance and comparing latent factor means within a confirmatory factor analysis framework. *Journal of Psychoeducational Assessment*, *29*(4), 347-363. https://doi.org/10.1177/0734282911406661

Sherbourne, C. D., & Stewart, A. L. (1991). The MOS social support survey. *Social Science & Medicine*, *32*(6), 705-714. https://doi.org/10.1016/0277-9536(91)90150-B
